# Per- and Polyfluoroalkyl Substances: Impacts on Morphology, Behavior and Lipid Levels in Zebrafish Embryos

**DOI:** 10.3390/toxics12030192

**Published:** 2024-02-29

**Authors:** Janice Albers, John Mylroie, Ashley Kimble, Catherine Steward, Kacy Chapman, Mitchell Wilbanks, Edward Perkins, Natàlia Garcia-Reyero

**Affiliations:** 1 Oak Ridge Institute for Science and Education, Environmental Laboratory, US Army Engineer Research & Development Center, Vicksburg, MS 39180, USA; 2 Environmental Laboratory, US Army Engineer Research & Development Center, Vicksburg, MS 39180, USAnatalia@icnanotox.org (N.G.-R.); 3 Bennett Aerospace, Raleigh, NC 27603, USA

**Keywords:** PFOA, PFHxS, PFOS, PFAS, lipids, lipidomics, behavior, zebrafish

## Abstract

The presence of per- and polyfluoroalkyl substances (PFASs) in aquatic environments is often persistent and widespread. Understanding the potential adverse effects from this group of chemicals on aquatic communities allows for better hazard characterization. This study examines impacts on zebrafish (*Danio rerio*) embryo physiology, behavior, and lipid levels from exposure to perfluorooctanoic acid (PFOA), perfluorohexane sulfonate (PFHxS), and heptadecafluorooctanesulfonic acid (PFOS). Embryos were exposed to lethal and sublethal levels of each chemical and monitored for alterations in physiological malformations, mortality, lipid levels, and behavior (only PFOA and PFHxS). The predicted 50% lethal concentrations for 120 hpf embryos were 528.6 ppm PFOA, 14.28 ppm PFHxS, and 2.14 ppm PFOS. Spine curvature and the inability of the 120 hpf embryos to maintain a dorsal-up orientation was significantly increased at 10.2 ppm PFHxS and 1.9 ppm PFOS exposure. All measured 120 hpf embryo behaviors were significantly altered starting at the lowest levels tested, 188 ppm PFOA and 6.4 ppm PFHxS. Lipid levels decreased at the highest PFAS levels tested (375 PFOA ppm, 14.4 PFHxS ppm, 2.42 ppm PFOS). In general, the PFAS chemicals, at the levels examined in this study, increased morphological deformities, embryo activity, and startle response time, as well as decreased lipid levels in 120 hpf zebrafish embryos.

## 1. Introduction

Poly- and perfluoroalkyl substances (PFASs) are a large group of fluorinated compounds that have a wide variety of commercial and industrial applications ranging from use in firefighting foams to non-stick coatings to fishing lines [1,2,3]. Decades of use and easy environmental transport in aquatic matrices has resulted in widespread contamination of soils and waters which is compounded by the environmental persistence of many PFASs or their breakdown products [4,5]. Once environmental contamination occurs, PFASs can bioaccumulate in individual organisms which can result in subsequent trophic magnification [6,7,8,9,10]. Exposure to PFASs can cause acute and/or chronic toxic effects in humans and other animals [6,11]. Specifically, PFAS exposure can have negative effects on development, growth, reproduction, hepatic function, immune function, neurological function, and lipid metabolism in humans and other vertebrates [6,11,12,13,14,15].

Zebrafish (*Danio rerio*) is a widely used model organism for studying vertebrate toxicology, and modifications of the original OECD Fish Embryo Acute Toxicity (FET) Tests have been utilized extensively as a high-throughput screening method that can be applied to other vertebrates, including humans [16,17,18,19,20,21]. The effects of PFAS exposure on zebrafish embryos have been studied in previous research with some common non-lethal effects including altered spontaneous tail bends, altered heart rate, un- or underinflated swim bladder, hatching rate, spinal/tail curvature, pericardial edema, cranial malformations, and tissue necrosis [22,23,24,25,26,27,28,29,30,31,32,33,34,35]. Altered visual motor response (VMR) behavior in PFAS-exposed embryos has also been seen across multiple exposures to different PFASs, with both hyper- and hypoactivity being observed in comparison to controls [23,25,28,30,36,37,38,39].

The liver is a target organ for PFAS accumulation in vertebrates which can result in disrupted lipid metabolism and ultimately hepatoxicity [15,40,41,42,43,44,45]. Previous metabolite profile and gene expression studies in zebrafish have identified perturbation in lipid metabolism as a result of PFAS exposure [27,29,42,46,47,48,49,50,51]. Specifically, many studies have focused on disruption of the peroxisome proliferator activating receptor (PPAR) pathways in zebrafish adults and embryos as a key response to PFAS exposure which can result in disruptions to lipid metabolism [29,49,52]. However, until recently there were no metabolomic/lipidomic studies on how zebrafish respond to PFAS exposure. These recent studies were limited in the number of PFASs tested but did find impacts on the metabolomic/lipidomic components [46,51]. Therefore, in an effort to better understand the specific lipid species that are affected by PFAS exposure and to better understand how these changes co-manifest with specific physical changes in zebrafish, we conducted 120 h exposures in zebrafish embryos to lethal and sublethal concentrations of PFOS, PFOA, and PFHxS. From these exposures, we determined the effects on development, morphology, and lipid levels for all three PFASs examined, and VMR behavior was assessed for PFOA and PFHxS.

## 2. Materials and Methods

### 2.1. Analytical Chemistry

Perfluorooctanoic acid (PFOA; CAS no. 335-67-1; >97% purity; Product #: 2121-3-18, Lot #: 00017912; Molecular Weight (MW): 414 g/mol; Water solubility: 9.5 g/L [53]) was obtained from Synquest Laboratories (Alachua, FL, USA). Tridecafluorohexane-1-sulfonic acid potassium salt (PFHxS; CAS no. 3871-99-6; Product #: 50929, Lot #: BCCD2880; MW: 438.20 g/mol; Water solubility: 1.4 g/L [54]) and Heptadecafluorooctanesulfonic acid potassium salt (PFOS; CAS no. 2795-39-3; >98% purity; Product #: 77282, Lot #: BCCC4690; MW: 538.22 g/mol; Water solubility: 0.68 g/L [55]) were obtained from Sigma-Aldrich (Saint Louis, MO, USA). Chemical concentrations were determined based on preliminary mortality range-finding tests in order to guarantee lethal and sublethal effects. For PFOA exposures, a stock solution of 500 ppm (mg/L) was made directly in E2 Media [56] by magnetically stirring the solution overnight in the dark, and subsequent nominal concentrations of 375, 350, 300, 250, and 200 ppm (≈905.8, 845.4, 724.6, 603.9, and 483.1 µM) were made in E2 media from the stock solution and buffered with 1N NaOH to a pH to match the E2 media (≈7.3). For PFHxS exposures, a stock solution of 50 ppm was made directly in E2 media by magnetically stirring the solution overnight in the dark, and subsequent nominal concentrations of 17.5, 15.0, 12.5, 10.0, and 7.5 ppm (≈39.9, 34.2, 28.5, 22.8, and 17.1 µM) were made in E2 media from the stock solution. For PFOS exposures, a stock solution of 100 ppm was made directly in Milli-Q water by magnetically stirring the solution overnight in the dark, and subsequent nominal concentrations of 2.0, 1.75, 1.5, 1.25, and 1.0 ppm (≈3.7, 3.3, 2.8, 2.3, and 1.9 µM) were made in E2 media from the stock solution. E2 media was used as the control solution for all three exposures.

All PFAS analytes, native and isotopically labeled, were purchased as mixtures at 1 µg/mL from Wellington Laboratories, Inc. (Guelph, ON, Canada). A secondary source of PFAS analytes for quality assurance purposes was purchased from Absolute Standards Inc. (Hamden, CT, USA). Ammonium acetate (99.99%) for mobile phase additives and LCMS grade methanol used for extraction and mobile phases were purchased from Sigma Aldrich (St. Louis, MO, USA). Optima (mass spectrometry, MS) grade acetonitrile for use as mobile phase and extraction was purchased from Fisher Scientific (Hampton, NH, USA). LC-MS grade water was purchased from Honeywell (Charolette, NC, USA) for use as mobile phase.

Both exposure media and embryo tissue samples were analyzed for PFOA, PFOS, and PFHxS concentrations. Exposure media samples were taken immediately before exposure with 250 mL collected for the control solutions and 7 mL collected for the PFOA, PFOS, and PFHxS solutions. To quantify PFAS concentration, a volume of methanol (MeOH), equal to that of the sample, was added in the original collection vessel. Methanol was added to the samples and diluted as needed to fall within the instrument’s linear range for each PFAS. In the final dilution, the internal standard was set at 0.7 µg/L. The sample was then put in a solution of 50:50 (MeOH:H_2_O) for analysis by LC-QqQ-MS. All samples were analyzed using an Agilent 1290 Infinity Binary Pump LC (Santa Clara, CA, USA) coupled to an Agilent 6495B triple quadrupole MS/MS with Jet Streaming Technology and electrospray ionization (ESI). Chromatographic separation was performed using an Agilent Poroshell 120 EC-C18 column (2.1 × 100 mm, 1.9 µm). An Agilent Eclipse Plus C18 RRHD column (3.0 × 50 mm, 1.8 µm) was used to delay any possible PFAS that is inherently in the system. Data acquisition was performed in dynamic multiple reaction monitoring (dMRM) mode using negative-mode ESI. Chromatographic separation was achieved by gradient elution with a flow rate of 0.4 mL/min, using 5 mM ammonium acetate in LC-MS grade water as mobile phase A and 5 mM ammonium acetate with 20% acetonitrile in MeOH as mobile phase B. The analytical column was held at a temperature of 50 °C during separation.

Embryo tissue accumulation was quantified from 3 pools of embryos made up of all the live embryos of each concentration collected from the two separate 48-well plates. The solvent extraction method used for this study was modified from a previous method [32]. Tissue samples were extracted in their original collection tubes and wet weights were collected before extraction. Prior to analysis, 0.42 ng of labeled analytes, used as surrogates, were added to all samples. Solvent extraction was completed by adding 300 µL of 70:30 acetonitrile and water to the tissue samples which were then sonicated for 18 h at room temperature. After sonication, samples were centrifuged at 14,000 rpm for 10 min, and the supernatant was transferred to a separate container and stored at −20 °C until analysis. Extracts were diluted with MeOH, and 0.21 ng of internal standard mix was added for analysis by LC-MS/MS. The limit of detection (LOD) was 20 ng/L for the media and 0.6 ng/g for the tissue samples.

### 2.2. Zebrafish Husbandry and Exposure

Wild-type, AB strain, adult zebrafish (Zebrafish International Research Center; Eugene, OR, USA) were housed on a Stand-Alone ZebTEC fish rack with Active Blue Technology (Tecniplast, West Chester, PA, USA) in tanks filled with conditioned reverse osmosis (RO) water and maintained at 28.5 °C with a 14 h:10 h light/dark cycle. For the conditioned RO water, the target water quality parameters were a conductivity of 750 µS/cm and a pH of 7.5, and parameters were maintained by automated delivery of 30 g/L Instant Ocean salts for conductivity and 30 g/L Sodium Bicarbonate for pH to RO water by the ZebTEC rack. Adult fish were fed GEMMA Micro 500 fish food twice a day (Skretting, Tooele, UT, USA).

Embryos were generated for this study by breeding five males and five females each in 11 different breeding tanks to produce 11 discrete breeding events. Adult fish were separated by sex the afternoon before the breeding event, via a plastic divider, in a 1.7 L Slope Breeding Tank (Tecniplast, Buguggiate, Italy) filled with system water. The breeding tanks were placed on tables in a temperature-controlled chamber (target of 26 °C) and housed under static conditions overnight. At the onset of the light cycle in the chamber the next morning, the dividers were removed from each tank and the fish were allowed to spawn, undisturbed, for approximately 30 min. After the breeding period had elapsed, the fish were removed from the breeding tanks and returned to the fish rack. Embryos from each breed were collected by breeding event and counted. The embryos from only the eight most productive breeding events were then washed and surface sterilized following a modification of [57], involving an extra rinse step using E2 media following bleach neutralization.

**Figure 1:**
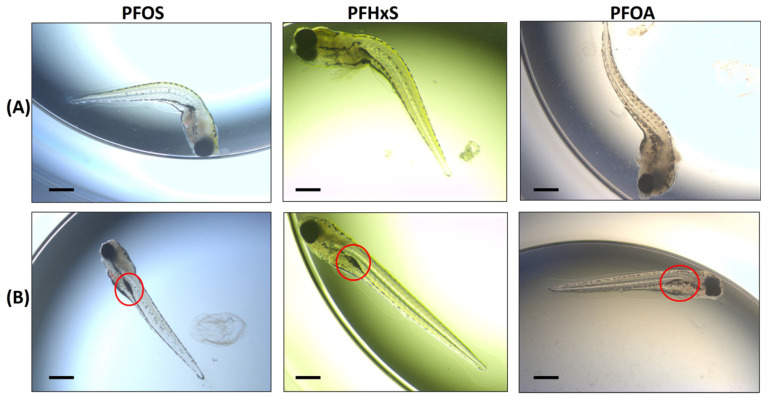
Examples of morphological endpoints assessed in zebrafish embryos. (**A**) Examples of the “Spine Curvature” endpoint among the 3 PFASs assayed (120 hpf embryos in images). (**B**) Examples of “Swim Bladder” endpoint among the 3 PFASs (120 hpf embryos in images). The red circle indicates position of the swim bladder which is un- or underinflated in each of the example images. Bar scale: 200 µM.

After washing, the embryos from each breeding event were screened for fertilization and divided into six batches of 24 embryos each. PFAS exposures were executed by using only one of the three PFASs per week. From ~7 hpf to 120 hpf, one of each of the six embryo batches was randomly exposed to one of the six PFAS (PFOA, PFOS, or PFHxS) concentrations for each of the eight breeds. Following batch exposure (which was the same concentration as the exposure solutions and lasted only for the duration it took to load the plates, i.e., ~1.5 h), the embryos were immediately placed in Falcon^®^ 48-well tissue-culture-treated plates (Corning Life Sciences, Corning, NY, USA), where each well contained 1 mL of fresh exposure solution. The embryos were reared in an incubator at a temperature of 28.5 °C with a 14:10 h light/dark cycle. For each of the 12 plates, the rows were randomized for PFAS concentration and the columns for breeding event (applied with a combination random and systematic column assignment; Figure S1). The night before the exposure, all plates were loaded with the correct PFAS solution/concentration and incubated overnight to reduce loss of PFAS via adsorption by the well plate walls. The following morning, solutions were discarded from the well plates and replaced with fresh solutions. The embryos were monitored by three technicians for physical malformations and mortality (Table 1; Figure 1), where technicians were not blinded to the treatment groups. Monitoring occurred once every 24 h with the exception being at 96 hpf: when PFOS exposed embryos could not be reliably assessed for swim bladder deformities. At 120 hpf, the movement behavior of exposed embryos was assessed using a VMR assay of 4 cycles of light and dark periods (see below). After the VMR assay, embryos were collected by pooling all the embryos from each concentration (plate row) for each plate in a 1.5 mL freezing vial (Product #: V4131, Sigma-Aldrich, Saint Louis, MO, USA), and then flash frozen using liquid nitrogen for subsequent tissue accumulation and lipidomics analysis.

### 2.3. Behavior Data Collection and Analysis

Visual motor response assays are commonly used to test the neurological system of fish by visually startling the fish and evaluating their reaction [58]. The change in light level is meant to startle the animal so that the reaction to that startle can be recorded. The VMR is similar to other behavior tests that use a percussion startle or touch startle. Our goal in using the VMR was not to assess what the animal did during the light levels, but how it physically responded to the visual startle. Reaction time to an environmental stimulus can be used, for example, to estimate how an animal may react in a predatory encounter; if too slow, then there can be adverse consequences. It can also provide some insights into how well the nervous system is performing.

In this study, VMRs were conducted using the DanioVision© Observation Chamber (Noldus Information Technology, Leesburg, VA, USA). Zebrafish embryos at 120 hpf were tested while in transparent 48-well plates containing ~1 mL of exposure solution. While in the testing chamber, embryos were isolated from light and sound, and experienced a constant temperature of 28.5 °C using the DanioVision© Temperature Control Unit. All VMR tests were conducted between 800 and 1500 h. The VMR test started after zebrafish embryos were positioned in a dark, pre-warmed behavior chamber and acclimated in the dark for 10 min (track data were not recorded during this period). After acclimation, fish movements were recorded while embryos underwent four cycles of alternating 3 min light and dark periods for a total assay length of 34 min (24 min of recorded movement data). The embryos in the VMR analysis experienced four startles of each type (four dark to light and four light to dark, for eight total startles), and eight 3-minute periods of differing light conditions: four dark and four light. Light levels during the light periods were set to 100% in the EthoVision XT© software, which is ≈2800 lx as measured by light meter. All embryos used in the VMR assay had also been previously assessed for morphometric deformities (see Table S1 for numbers of embryos). Due to logistical constraints, VMRs were not performed on embryos from the PFOS exposures.

Spontaneous movement of all embryos was constantly recorded at a rate of 30 frames per second and tracked using EthoVision© DanioVision© system version 11.5 (Noldus Information Technology, Leesburg, VA, USA). Software settings for tracking did not include smoothing of tracks or a minimal distance before movement recording. Before analysis was conducted, all embryos that were dead or had physical deformities that would severely impact movement (these included embryos with curved tail or spine, obviously un- or underinflated swim bladder, weak heartbeat, slow or non-detectable blood flow, and/or edemas) were removed from the analysis (Table S1). Embryos that displayed impaired equilibrium that was not obviously attributable to swim bladder malformations were included in behavioral analyses. After review of the video overlay with the raw tracking data, two main tracking errors were identified: (1) artificial movement between two parts of the fish and (2) large jumps to the edge of the arena. These two error types were corrected using a technique similar to that in [59]. Locations with the first error type were flagged using the following criteria: turning angle between previous and future location was >150 degrees and distance between previous and future location was <0.06 mm. During periods with high error occurrence, movement less than 0.2 mm was ignored. Locations with the second error type were flagged using the following criteria: turning angle between previous and future location was >160 degrees, distance between previous and future location was <=0.6 mm, and the distance traveled between the location and each of the previous and future locations was >=2 mm and <=30% difference in length. Once locations of errors were identified, all locations flagged as errors were replaced using equidistance locations between the nearest two non-error locations. Additionally, six embryos were removed from the VMR analysis due to EthoVision© either losing the fish location for multiple seconds or poor tracking due to inaccurate arena designation (Table S1).

Individual embryo activity was defined in each frame using the corrected fish locations. Swimming speed was calculated as mm per second and swimming distance traveled in mm. Swimming was defined as movement that was at least 6 mm/s or 0.2 mm per frame (i.e., magnitude of velocity at embryo’s center) and lasted longer than 5 frames (0.166 s). Resting occurred during frames where movement was less than 1 mm/s or, if greater than 1 mm/s, lasted less than 5 frames. When resting behavior was performed, the speed and distance for those frames were changed to zero for the analysis. In addition, the turning angle associated with each frame of swimming was calculated using the difference between the four-quadrant inverse tangent of the two trajectories, where the first trajectory was constructed from the first two locations in the sequence, and the second trajectory from the second two locations in the sequence. The resulting turn angle ranges from −3.14 to 3.14, where zero is straight ahead movement, negative values indicate right turns, and positive values indicate left turns.

For this study, we had two behavior areas of focus: (1) general overall effects of PFAS exposure and (2) specific startle response reaction times. To address the former, we calculated 10 different average behavior endpoints for each individual embryo during the VMR assay (see Table S2 for list and detailed descriptions). Each endpoint was summarized by averaging the performance of that behavior over the entire VMR assay. Types of behavioral endpoints included total distance traveled, total time swimming, overall average step length and variation, and overall turning angle and variation. Overall swimming bout characteristics (i.e., time between rest periods) were summarized using multiple metrics: number of bouts per second and the average bout duration, speed, and turning angle. To address the second behavior area of focus, we calculated four startle-specific behavior endpoints. Embryos in this study responded to the change in light during the VMR using the typical startle response pattern observed in previous studies (e.g., Emran et al., 2008 [58]). To determine differences in this startle response pattern, four behavior endpoints were calculated that were specific to how the embryos responded to the visual startle of the light turning off and on. (1) The average magnitude of the embryo response was determined first by identifying the maximum speed traveled within 5 s after the startle. Then the magnitude of the startle response was calculated using the difference between this maximum embryo speed and the embryo speed at the time of the startle. (2) The average time it took for the embryo to respond to the visual startle was calculated as the difference in time between the time of the startle and the time where the maximum speed was observed. (3) Average startle distance was defined as the distance the embryo swam between the startle time and the time of maximum speed. (4) Lastly, average distance after the startle was calculated by totaling the distance traveled during the 5 s after the startle.

### 2.4. Lipid Extraction and Analysis

As with the tissue collected for PFAS analysis, the embryos used for lipid extraction and subsequent analysis were pooled by individual plate and concentration (row) and then flash frozen at 120 hpf. For the PFOS exposure, both live and dead embryos were pooled together to have enough sample tissue for testing, but for the PFOA and PFHxS exposures, only live embryos were pooled. Each chemical had 5 pools collected for analysis (excluding a few exceptions in the higher concentrations), with the number of embryos in each pool ranging from 2 to 8 (Table S3).

Data acquisition and analysis were carried out at the Kansas Lipidomics Research Center using an automated ESI-MS/MS approach, as described previously [60] with a brief description to follow. Embryo pools were homogenized under frozen conditions and lipids were extracted after resuspension in water with a 1:3 chloroform-to-methanol ratio. The organic layer was saved, and the aqueous layer was re-extracted with chloroform three times with the organic layer retained and pooled. The pooled organic layer was washed with water, evaporated, and then re-suspended in chloroform, when 20 µL of the re-suspended sample was used for mass spectrometry.

Mass spectrometry analysis was conducted using a XEVO TQ-S (Instrument #: WAA627, Waters Corporation, Milford, MA, USA) with the injection consisting of the extracted sample, an internal standard mix (full details of standards used in Table S4), and chloroform/methanol/300 mM ammonium acetate in water in a 300/665/35 ratio for a final total volume of 1.2 mL. Samples were introduced to the electrospray ionization component of the triple quadrupole mass spectrometer from an autosampler at a rate of 30 µL/min. The spectral data were generated using sequential precursor and neutral loss scans of the introduced sample mix. Full parameters for spectral data generation can be found in the supplemental material (Table S5). Once spectra were acquired, a custom script, written in Visual Basic (Microsoft Corporation, Redmond, WA, USA) by Iggy Kass (Waters Corporation), was used in conjunction with the Waters Corporation software MassLynx (v4.2) to subtract the background from each spectrum, smooth the data, and integrate the peaks. A quantified list of lipids was then generated for each sample using the online analysis application LipidomeDB() Data Calculation Environment [61]. Seven quality controls (pooled samples) were analyzed among the other samples. If the coefficient of variation (i.e., standard deviation/average) for an analyte in the quality controls was more than 0.3, the data on that analyte were eliminated from the reported data. Lipids were normalized to wet weight of pooled embryos (mg), and values are presented as nmol per mg dry lipid weight. Each sample was examined for 16 types of polar lipids (355 separate compounds; Table S6), as well as totals of each lipid class.

### 2.5. Statistical Analysis

Multiple endpoints in the same individual (lipids, morphology, and behavior) can help researchers assess multiple physical attributes that may lead to theories about chemical modes of action. This also allows for greater efficiency with information collection while conducting an intensive/expensive study. However, from a statistical standpoint, observations from the same individual are not independent. Consequently, we constructed a one-way ANOVA nonparametric permutation test for each endpoint. Permutation ANOVA tests were conducted using the AOVP function in the lmperm package for R (“lmPerm” version 2.1.0, Bob Wheeler, Marco Torchiano, Torino, Italy) for permutation-based ANOVA [62]. Continuous endpoints, such as the behavior and lipid levels, were estimated using a linear response, while occurrence of physiological responses was estimated using a logistic response model (i.e., binomial response). For all tests, we required the estimated standard error of the estimated proportion *P* to be < *P* × 10^−5^ and the number of iterations set to 2 × 10^9^. Using these settings, the *p*-value varied by ≤0.0001 between runs of the model. Type III sums of squares are reported. Treatment level differences were examined by computing Tukey’s Honest Significant Difference (HSD) which uses a family-wise probability of coverages. By random chance, the number of ANOVA behavior tests that could be significant is ~2 out of the 28 behavior endpoints (0.05 alpha level × 28 ANOVAs = 1.4). Note: The Tukey HSD adjusts for the number of pairwise comparison tests performed within each ANOVA. Lastly, the 10th and 50th percent lethal dose was calculated using media concentrations and the lc function [63]. All analyses were performed using R version 4.0.4 [64]. Significance level was alpha = 0.05.

## 3. Results

### 3.1. Analytical Chemistry

Media concentrations of PFOA and PFHxS were, on average, 8.2 and 17.7% lower than the nominal concentrations, respectively, whereas PFOS media concentrations were 18.8% higher (Table 2). In all exposures, the control solution (E2 media) had PFOA, PFHxS, and PFOS concentrations below the limit of detection (<LOD); however, the control embryos contained low levels of the tested exposure PFAS (Table 2).

### 3.2. Morphometric Results

**Figure 2:**
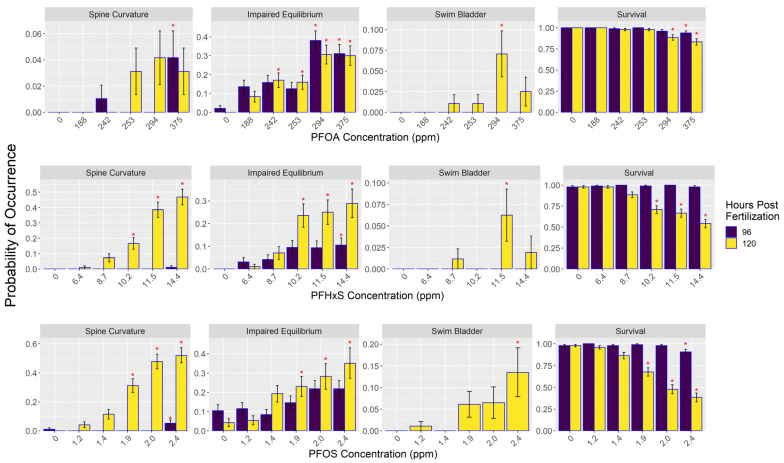
PFAS treatment impacts on the probability of occurrence for survival and morphological characteristics of 96 and 120 h post-fertilization zebrafish embryos. PFAS concentrations are from the media used in each treatment. Red asterisk indicates significant difference between treatment and control groups. Error bars represent estimated standard deviation assuming normal distribution. See Table S1 for the number of fish in each treatment.

PFAS exposure resulted in significant effects on physical morphology and survival of embryos (Figure 2; Tables S7 and S8). Significantly decreased survival was found in 120 hpf embryos starting at 294 ppm for PFOA, 10.2 ppm in PFHxS, and 1.9 ppm in PFOS. At 96 hpf, survival decreased at 375 ppm in PFOA and 2.4 ppm in PFOS, whereas there was no significant effect on survival in PFHxS in the 96 hpf embryos. Predicted lethal concentration of 10% (LC10) of the 120 hpf embryos was 318.1 ppm for PFOA, 8.6 ppm for PFHxS, and 1.41 ppm for PFOS (Tables S10 and S11). Predicted lethal concentration of 50% of the 120 hpf embryos (LC50) was 528.6 ppm for PFOA, 14.28 ppm for PFHxS, and 2.14 ppm for PFOS. The lethal concentration at 50% determined using tissue concentrations (LC_t_50) was 124 ppm for PFHxS and 2030 ppm for PFOA; LC_t_50 for PFOS was 850 ppm but the model that generated this estimate had high levels of uncertainty.

PFOA above 242 ppm increased the occurrence of 120 hpf embryos’ inability to correct their equilibrium and orientate their body in a dorsal-up position as measured by the term impaired equilibrium. This effect was also observed in PFHxS starting at 10.2 ppm and at 1.9 ppm in embryos exposed to PFOS (Figure 2; Tables S7 and S8). An increase in the occurrence of spine curvature was observed at 120 hpf in embryos exposed to PFHxS concentrations above 10.2 ppm and PFOS concentrations starting at 1.9 ppm. PFOS exposure at 2.4 ppm increased swim bladder malformations.

### 3.3. Behavior (Visual Motor Response)

**Figure 3:**
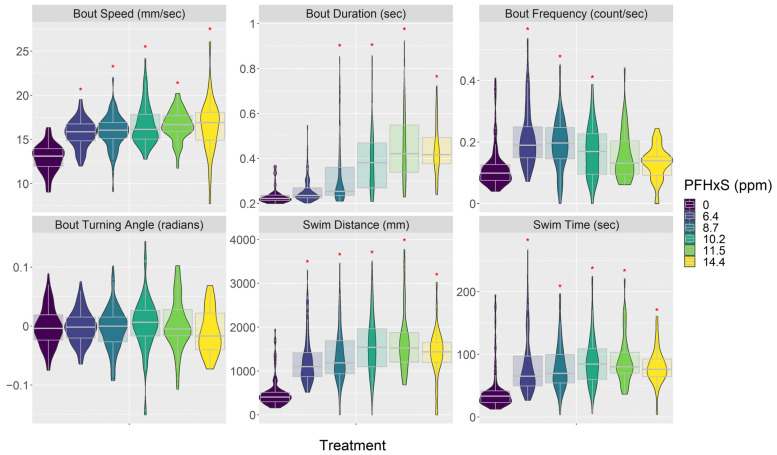
PFHxS treatment impacts on embryo behavior. See supplemental material for all behavior response plots and model results. PFHxS concentrations are from the media used in each treatment (Table 2). Red asterisk indicates significant difference between treatment and control groups. Data are presented as violin plots overlaid with the 95th percentile box plot. See Table S1 for the number of fish in each treatment.

**Figure 4:**
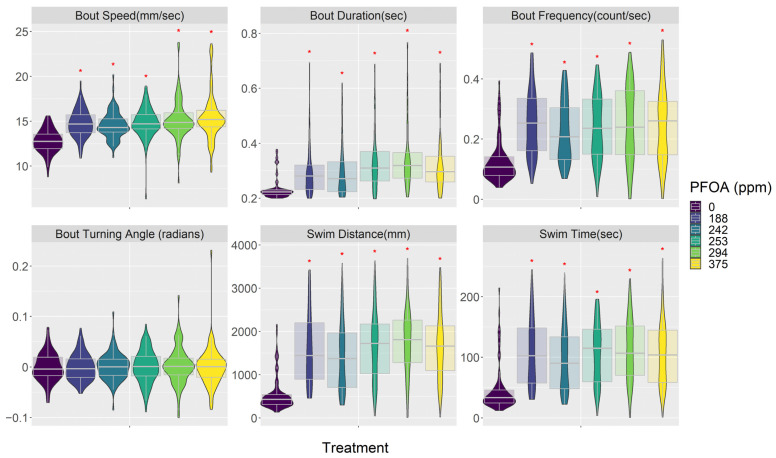
PFOA treatment impacts on embryo behavior describing the response of swimming bouts and activity levels. See supplemental material for all behavior response plots and model results. PFOA concentrations are from the media used in each treatment (Table 2). Red asterisk indicates significant difference between treatment and control groups. Data are presented as violin plots overlaid with the 95th percentile box plot. See Table S1 for the number of fish in each treatment.

Both PFOA and PFHxS affected embryo behavior in similar ways (Figure 3, Figure 4 and Figures S2–S5; Tables S7–S9). In general, they made embryos swim faster and increased their activity but also decreased their response to visual stimuli; these reactions were consistent across most of the concentrations tested. PFOA and PFHxS increased swimming bout speed, duration, and frequency. PFOA and PFHxS also increased step length, total distance traveled, and time swimming. Additionally, all levels of PFOA and PFHxS in this study increased time of reaction to a startle, magnitude of startle response, and distance traveled before and after the startle. Lastly, PFOA and PFHxS decreased the variability in turning angles the fish swam. Most of these significant relationships exhibited linear increases as PFOA and PFHxS concentrations increased, with the exception being the frequency of swimming bouts, which responded in a curvilinear pattern as PFHxS concentrations increased. Swimming bout frequency was significantly increased compared to the control treatment at the three lowest PFHxS concentrations, with the highest observed frequency occurring at 6.4 ppm and generally decreasing as the PFHxS concentrations increased (Figure 3). The only behavior endpoint not affected by either PFOA or PFHxS at the concentrations tested was the mean turning angle each fish swam.

### 3.4. Lipidomics

PFOA, PFHxS, and PFOS decreased levels of 35 phospholipids, while PFOS also increased seven fatty acids (FA; Table 3, Table 4 and Table 5 and Tables S7–S9). Only one phospholipid was significantly altered by all three PFAS chemicals tested in this study, phosphatidylserine (40:5) (PS), which was lowered at 294 ppm PFOA, 14.4 ppm PFHxS, and 2.03 and 2.42 ppm PFOS treatments. Both PFOA and PFHxS decreased the amount of total ether-phosphatidylcholine (ePC) lipids in the 294 and 375 ppm PFOA, and 14.4 ppm PFHxS treatments. PFOA’s impacts on phospholipids were mainly on phosphatidylserines (PS) but only in a dose-dependent manner for PS(34:1) and total ePC (Table 3). PFHxS altered nine different phospholipids in the highest PFHxS treatment, 14.4 ppm, but only PS(38:4) was also altered in any lower PFHxS treatment (Table 4). The lack of dose-dependent response in the majority of the phospholipids after PFHxS exposure suggests more testing is needed to make sure the lower number of embryos in the higher PFHxS treatments did not affect these results (Table S3). Lastly, phosphatidic acid (34:1) (PA) had a consistent negative trend with PFOS concentrations above 1.4 ppm. Concentrations at and above 2.03 ppm PFOS also decreased phosphatidylethanolamine (38:5) (PE), PE(42:8), PS(40:5), and PS(40:6) (Table 5). Overall, 2.42 ppm of PFOS altered 20 different phospholipids, with only five phospholipids having significant changes in more than just the highest treatment. Again, this suggests that more testing is needed to make sure the sample quality and number of the embryos in the highest PFOS treatments did not affect these results.

## 4. Discussion

Comparing the lethal media concentrations found in this study to previous work, the PFHxS media LC50 of 14 ppm (mg/L) found in this study is ~10 times lower than the PFHxS LD50 of 134 ppm reported in Annunziato et al. [22], on 120 hpf zebrafish embryos. The PFOS media LC50 of 2.14 ppm found in this study is similar to the media LC50 of 2.25 ppm previously reported [29] and the LD50 of 2.20 ppm on 120 hpf zebrafish [25]. However, the PFOS media LC50 of 2.14 ppm found in this study was slightly lower than the PFOS dose LC50 range of 3.5–81 ppm summarized previously for embryo cyprinids [6]. The PFOA media LC50 of 528 ppm from this investigation is within the previously reported range of PFOA dose LC50 of 561 ppm in zebrafish embryos and 24–759 ppm for cyprinid embryos [6,34]. Commonly not measured and reported in studies, the LC_t_50s found in this study were 3.8, 8.7, and 397 times higher than the media LC50s for PFOA, PFHxS, and PFOS, respectively (Table S10). This suggests that PFAS metabolism and bioaccumulation are important aspects that may be missed when reporting only media or dose level LC50s.

The reasons for high variability in PFAS LC50s are a point of active research, with causes ranging from varying laboratory practices [66] to environmental factors, such as embryo age and water chemistry, as probable causes of variation in LC50 values. For example, research has found PFAS LC50s from zebrafish embryos are negatively correlated with increasing embryo age at assessment, possibly from delayed mortality due to developmental liver toxicity [42,67]. Embryo age was compensated for in our comparisons by only using LC50s from 120 hpf zebrafish embryos. Another factor that may influence the LC50 results is media pH. Unbuffered PFOA media results in a 96 hpf LC50 that is ~10 times lower than buffered PFOA media (used in this study), with unbuffered PFAS solutions of ≥25 ppm having pH ≤ 5.54 [33]. Another factor that likely influenced the LC50s reported in this study is that the PFOA concentrations tested in this study were not high enough to result in 50% mortality. Consequently, our estimates of PFOA LC50 are based on model predictions assuming a probit curve. Lastly, PFASs can easily bioaccumulate in laboratory embryo tissues, resulting in fish tissue concentrations that are substantially higher than media concentrations (Table 2; e.g., [38]). Any laboratory condition or method that influences the bioaccumulation rate could dramatically alter the embryo PFAS tissue levels and consequently the LC50s. Reporting LC50s based on tissue concentrations in parallel with media concentrations would allow for more accurate comparisons of lethal impacts from PFAS [32]; however, metabolism of PFAS and gender differences may confound this technique.

All three PFAS chemicals in this study altered the ability of 120 hpf zebrafish embryos to orient themselves in a dorsal-up position, with this endpoint first manifesting at 242 ppm PFOA, 10.2 ppm PFHxS, and 1.86 ppm PFOS. These impairments to equilibrium were only partially due to air bladder deformities since these had only been significantly increased at 294 ppm PFOA, 11.5 ppm PFHxS, and 2.4 ppm PFOS. Significant increases in spine curvature coincided with the embryos’ inability to right themselves, at 96 hpf for PFOS and PFOA, and at 120 hpf in PFOS and PFHxS exposures. The increased rates of impaired equilibrium found in this study did coincide with LC10s estimates for all three chemicals, suggesting the impaired equilibrium responses observed in this study may be linked to a pre-mortality stage. Studies have shown that multiple PFASs, including PFOA, increased listing percentage in zebrafish embryos [42,68], and this endpoint has been used to assess non-PFAS chemicals as well [69,70]. Additionally, Chen et al. [71] showed that PFOS created equilibrium issues more often than pericardial and yolk sac edemas, at a similar incidence percentage as uninflated swim bladders, and less often than malformed tails. Even with these limited investigations, there is strong evidence from other research that PFAS compounds affect vertebrate brain functions such as calcium homeostasis, neuronal signaling, and release of neurotransmitters, which could be a contributing factor for the observed impairment of the embryo equilibrium in this study [72,73]. In addition, there are data from other studies showing that sublethal doses of PFOS, PFOA, and PFHxS affect multiple aspects of embryo morphology [42,71,74,75,76]. However, more research is needed to understand how low levels of PFOS, PFOA, and PFHxS affect developmental neurology and morphology, and how these developmental impacts may be linked to the inability of embryos to maintain equilibrium.

Similar to the results found in this study, previous research into the behavioral effects of PFOA exposure on zebrafish embryos have found it to increase activity [26,34,38,39,74]; however, multiple studies have found no change or reduced activity at PFOA levels similar to those examined in this investigation [73,77,78], or even lower, more environmentally common, PFOA levels [28,47]. In this study, all PFOA exposure concentrations increased activity, with the lowest concentrations tested being 188 ppm. Previous studies have found activity increases at much lower PFOA levels, such as 0.08, 0.4, 4.7, and 5 ppm [26,34,38,74]. In contrast, previous research on the effects of PFHxS exposure on zebrafish swimming behavior have been mixed. In this study, PFHxS increased zebrafish embryo activity at all levels, starting at 6.4 ppm. Gaballah et al. [23] also found hyperactivity in some light and dark periods of the VMR starting at 1.76 and 5.6 ppm. However, two studies found PFHxS to decrease activity at 4.8 and 8 ppm [22,38], and Rericha et al. [39] found no activity change at 0.228 ppm PFHxS. Therefore, even though there is general agreement from previous studies that that high levels of PFOA increase zebrafish embryo activity, there are conflicting results as to how lower concentrations of PFOA and both higher and lower concentrations of PFHxS impact embryo activity. Multiple aspects could be contributing to the conflicting results, including best PFAS laboratory management practices [66] or uncorrected fish tracking errors (noted in Section 2 above). Additionally, recent research of generational effects of PFOA on zebrafish behavior indicated that PFOA exposure can increase or decrease activity depending on the generation assayed, and thus, showed that previous lineage exposure can also affect behavior [47]. Future replication and tailoring of PFAS toxicological studies will be needed to discern overall behavior trends from these chemicals.

Visual motor response fish behavior assays are commonly applied in toxicological assessments [79]; however, possibly due to software limitations, the fish’s response to the visual startle of the light-to-dark or dark-to-light change is not often analyzed. In this study, both PFOA and PFHxS increased embryo reaction time, the magnitude of the startle response, and the distance traveled after the startle (Figures S2 and S5). These results indicate the embryo’s ability to sense the environment is delayed after PFOA and PFHxS exposure, and once the embryos respond, their reactions are increased in magnitude and activity. This may indicate that in real predatory encounters, embryos exposed to PFOA or PFHxS would have a delayed response and possibly overreact to the predator. Of the eight previous studies that conducted VMRs after PFOA and PFHxS exposure, only Menger et al. [38] analyzed the specific startle response of exposed fish to the change in light and found an increase in the embryo startle response at 4.8 ppm PFHxS. Two other studies mention the startle response being altered by PFAS exposure but did not measure it specifically, with both studies finding PFOA decreasing the startle response at 165.6 and 10–1000 ppm [73,78]. As is typical of most toxicological studies, the remaining five only examined activity level differences between the dark and light periods of the assay [23,26,39,74,77]. Combining these results with the current study’s results suggests levels of PFHxS 4.8 ppm or greater will reduce an embryo’s ability to respond to external stimuli. In addition, PFOA at concentrations starting at ≈165 ppm will alter an embryo’s ability to detect external stimuli, but it is unclear in which direction the change occurs. The response time of embryos to different possible neurotoxicants could be an important measure of an embryo’s ability to sense their environment. Since there is already widespread use of the VMR assay in toxicology, the addition of a startle response endpoint calculation to the standard methodology would benefit researchers in their ability to understand how chemicals affect “real world”-type behavior endpoints.

The inclusion of multiple types of omics is contributing to our understanding of how pollutants affect biological systems [80,81]. Lipidomics could play a major role in understanding how pollutants, such as PFASs, impact metabolism [46,82,83,84], but baseline knowledge into how lipids change over zebrafish embryonic development is still an active area of research [51,85]. There is mounting evidence that fish lipid metabolism is altered by PFASs. For example, PFOS alters embryonic nutrition and pancreatic morphometry [85,86], and induces severe fatty degenerations in male livers [87]. PFHxS causes oxidative stress, inflammation, and impaired fatty acid β-oxidation [51], and PFOA creates dysfunctions of carbohydrate, lipid, and amino acid metabolism [42]. Results from this study indicate PFOA, PFHxS, and PFOS decreased multiple lipid types in zebrafish embryos with mainly complex lipids being affected, such as phospholipids including PE, PC, and PS. Interestingly, PFOS (but not PFOA or PFHxS) also increased several types of simple lipids like FAs. The only phospholipid found in this study to be lowered by all three PFAS chemicals was PS(40:5). PS is critical for coagulation and required for healthy nerve cell membranes and myelin, which supports cognitive functions mainly in mammals [88], but derivatives of PS are also present in Archaea and macrophages [89]. PS signaling and recognition by receptors are essential for normal zebrafish embryo development [90] and studies have shown that dysregulation of PS levels can be associated with morphological malformations similar to those seen in this study. Shibata et al. [91] found that downregulation of lysophosphatidylglycerol acyltransferase 1 resulted in decreases in various phospholipids, including PS, that coincided with developmental malformations in zebrafish, including bent embryo axis (spinal curvature). Dysregulation of phosphatidylserine synthase (PSS), which catalyzes the production of PS, has been linked to skeletal malformations in humans and severe angulation of the trunk (spinal curvature) in zebrafish [92]. Furthermore, disruption of the PSS gene caused developmental defects and impaired neurological function in *Drosophila melanogaster*, indicating that beyond the morphological malformations seen here, altered PS levels may have an effect on behavior [93].

Both PFOA and PFHxS were found to decrease the amount of total ePC lipids at 294 and 375 ppm PFOA and 14.4 ppm PFHxS. ePCs play a role in inflammation, are important membrane constituents of neutrophils, and play a role in the immune response of animals, plants, and even bacteria [89,94]. Moreover, ether phospholipids (such as ePC) have been implicated in having a potential role in behavior and cognition [95], and a study using KO mice for the glyceronephosphate O-acyltransferase gene, which is essential for the synthesis of ether phospholipids, displayed hyperactivity and other impaired behaviors [96]. This connection between ether phospholipids and behavior may lend insight into the mechanisms connecting PFAS exposure in zebrafish embryos and altered VMR behavioral response; however, more research on this potential connection is necessary before any definitive conclusions may be made.

As an overall trend in zebrafish embryos, exposure to PFOS, PFOA, and PFHxS decreased phospholipids, which are the building blocks of cellular membranes in all living organisms [85,89]. However, in this study, each PFAS examined impacted a different combination of phospholipids. For example, PFOS decreased multiple types of glycerophospholipids, 1-PA, 1-ether-phosphatidylethanolamines (ePE), 2-PSs, and 7-PEs. Specifically, PA is critical for the metabolism of other phospholipids in animal and plant cells and plays an important role in cell signaling using lipid-gated ion channels [89]. PEs, on the other hand, are abundant in the nervous system and can be found in brain white matter, neural tissue, and the spinal cord [89]. Studies have demonstrated that PFAS can disrupt PPAR signaling [15,51], and the decrease in PE species in PFOS and PFHxS exposed could be due to PFAS-induced PPAR downregulation as inhibition of ppar γ has previously been shown to decrease PE species in zebrafish embryos relative to controls [97]. PEs play a role in the mechanism of cellular signaling and are generally less abundant in animal and plant cells but are the principal phospholipid in bacteria [89]. ePEs are the second most abundant phospholipid in animal liver and brain tissue, found in higher proportions in mitochondria than other organelles, and are typically the main component of microbial membranes. This lipid is an essential component in the cell membrane’s ability to form membrane fusion structures (lipidmaps.org). PFOS has been shown to decrease multiple types of phospholipids in mouse kidney, including PAs and PEs [98], and this may be due to the degradation of the membrane protein components, resulting in membrane leakage and variation in rigid structure [99,100,101]. One recent study using injection of PFOS into zebrafish embryos at levels equal to and lower than those in this study found very similar results, with Yang et al. [85] showing downregulation of PE and PCs in zebrafish embryos after PFOS exposure.

This study also found that PFOS increased seven fatty acids and decreased multiple phospholipids. Fatty acids are a source of energy in tissues and provide rigidity in membranes [89]. The increase in fatty acids after PFOS exposure found in this study agrees, in general, with previous research on mammals [6]. However, Arukwe et al. [102] found PFOS decreased fatty acids in Atlantic salmon larvae (*Salmo salar*), with some of the same fatty acids decreased in Atlantic salmon found to be increased in zebrafish in this study (FA(16:0), FA(18:1), FA(20:5), FA(20:1), FA(22:6)). As previous mammalian research suggests, fatty acid increase after PFOS exposure could be due to oxidative stress or activation of peroxisome proliferator-activated receptors resulting in severe steatosis [6], and Yang et al. [85] also found fatty acids were upregulated in zebrafish embryos after PFOS exposure.

Also observed in this study was that PFOA decreased 1-ePE, 1-dihydrosphingomyelin (DSM), 2-PEs, 6-PSs, and total lyso-phosphatidylethanolamine (LPE) and ePCs, but only in a dose-dependent manner for PS(34:1) and total ePCs. DSM is a type of sphingomyelin (SM) found in animal cell membranes, especially in the membranous myelin sheath surrounding some nerve cell axons. SMs play a role in signal transduction and DSM is essential to eye lens formation [89,103]. LPE is a minor component of cell membranes and plays a role in cell signaling, activation of other enzymes in animals and plants, and membrane lipid degradation in plants [104]. PFOA has been shown to cause hypolipidemia due to independent and dependent PPAR mechanisms through inhibition of PC synthesis via demethylation of PEs [105]. Yang et al. [85] also found the major class of lipidomic dysregulation after PFOA was the downregulation of PE and PC lipids in zebrafish embryos.

Lastly, this study found PFHxS altered a combination of multiple types of phospholipids previously mentioned (2-ePCs, 2-PCs, 2-PEs, and 2-PSs) in the 14.4 ppm PFHxS treatment, but only PS(38:4) was altered in any lower PFHxS treatment. Xu et al. [51] also found PFHxS to alter 120 hpf zebrafish lipids, with the main perturbations occurring in PC and PE associated with defense mechanisms and with ether-lipids and lyso-phosphatidylcholines that are related to oxidative stress and inflammation. Yang et al. [85] saw similar disruptions to PC and PE levels in zebrafish embryos exposed to PFOA and PFAS and indicated that this dysregulation could result in oxidative stress with impacts on developmental malformations similar to those seen in this study. Perturbations in PC levels have been shown to effect proper cognitive function [106], and oxidative stress and reactive oxygen species have been linked to impaired swim bladder inflation in zebrafish exposed to PFAS and other toxic chemicals [107,108]. Furthermore, PFASs have been implicated in disruptions to proper thyroid function, which can affect proper swim bladder formation and disrupt lipid metabolism [31,109,110,111]

While this study found PFOS, PFOA, and PFHxS to generally decrease levels of lipids in 120 hpf zebrafish embryos, the impacts on lipids in developing zebrafish embryos from these chemicals are complex. With some studies reporting increases in lipid levels and others reporting decreases, understanding of how PFAS is affecting biological functions remains unclear. However, the combination of the results from this study and the recent study by Yang et al. [85], both containing very similar lipidomic results (even with minor exposure methodological changes), provides good evidence for the types of lipids that PFOS and PFOA exposure are changing. These changes in lipid content have potential associations to the apical malformations and behavioral changes seen in this study. However, in addition to differing levels of exposure, multiple biological functions are taking place such as oxidative stress, inflammation, and defense mechanisms, as well as naturally changing levels of lipids depending on the development stage [51]. Consequently, much more knowledge is needed about natural and altered lipid levels to understand the full impacts of PFAS exposure on developing zebrafish and the relationship between altered lipid levels and the endpoints observed in this study. Lastly, many challenges to PFAS laboratory methodology and exposure are still being explored, such as the lack of samples in the higher PFAS doses influencing the lack of dose-dependent response in the majority of the phospholipids. All of these complexities suggest that additional studies are needed to fully understand lipidomic impacts from PFAS exposure in both zebrafish and humans.

## 5. Conclusions

In conclusion, this study found PFOA, PFHxS, and PFOS all impacted the development of zebrafish embryos. At the five dose levels tested for each chemical, we found all three PFASs impacted morphology, including increasing the occurrence of spine curvature and inability to maintain a dorsal-up orientation. Additionally, behavior tests on PFOA- and PFHxS-exposed embryos showed that treatments made fish swim faster and have increased activity, but embryos were less responsive to visual stimuli. In the highest levels tested, this study also found whole body decreases in certain lipids after all three PFAS exposures. These impacts can be used to better understand the lethal and sublethal impacts PFAS pollutants have on fish embryos and how alterations in lipid species may be associated with those impacts. Since zebrafish are a model species, these results can be related to human health impacts as well.

## Figures and Tables

**Table 1:** Description of morphological endpoints collected in this study.

Morphological Endpoint	Definition
Mortality	Lack of heartbeat or obvious necrosis or ruptured pericardium.
Spine Curvature	Curvature of the spine/upper tail similar to the scoliosis phenotype described in von Hellfeld et al. (2020) [21]. Documented on live and dead embryos.
Swim Bladder	Incidences where the swim bladder was either underinflated or uninflated. Documented on live embryos.
Impaired Equilibrium	Embryos that were either on their side or had difficulties staying in the upright or “dorsal-up” position, which can also be described as “listing”. Documented on live embryos with no external stimulus applied.

**Table 2:** Media and tissue concentrations of tested PFASs found in the treatments of this study. Tissue concentrations were determined using three pools of fish, containing various numbers of embryos. Std = standard deviation.

Chemical	Nominal Concentration ppm	Media Concentration ppm (% Recovery)		Embryo Tissue Concentration ng/mg (Std)		Average Number of Embryos in 3 Pools
PFOA	0	<LOD of 20 ng/L		12.24 (6.1)		16.0
	200	188	(94.0)	487	(85.9)	16.0
	250	242	(96.8)	263	(25.0)	15.7
	300	253	(84.3)	305	(10.4)	16.0
	350	294	(84.0)	361	(49.3)	15.3
	375	375	(100.0)	485	(50.6)	13.7
PFHxS	0	<LOD of 20 ng/L		0.2 (0.3)		16.0
	7.5	6.35	(84.7)	69.0	(20.9)	15.7
	10	8.70	(87.0)	91.5	(12.3)	13.7
	12.5	10.15	(81.2)	71.4	(7.0)	14.3
	15	11.50	(76.7)	76.0	(25.5)	11.3
	17.5	14.35	(82.0)	94.5	(13.6)	7.3
PFOS	0	<LOD of 20 ng/L		2.32 (2.8)		8.0
	1	1.21	(120.9)	148	(36.7)	8.0 ^a^
	1.25	1.40	(111.9)	227	(77.7)	8.0 ^a^
	1.5	1.86	(124.2)	197	(53.1)	8.0 ^a^
	1.75	2.03	(116.1)	183	(153.6)	8.0 ^a^
	2	2.42	(121.0)	103	(78.5)	8.0 ^a^

^a^ Included recently dead embryos.

**Table 3:** Summary of PFOA significant trends found using Tukey HSD on the lipidomics endpoints. Decrease or Increase indicates trend of accumulation of lipid group in control vs. treated sample. All lipid species shown in the table were significantly different in treated samples vs. the control at a *p*-value < 0.05 and an * indicates lipid species significant a *p*-value < 0.01. - indicates no significant pattern in lipid group accumulation in treated sample vs. control. See Fahy et al. (2009) [65] for detailed lipid acronym definition. PE, phosphatidylethanolamine; PS, phosphatidylserines; ePC, ether-phosphatidylcholine; ePE, ether-phosphatidylethanolamine; SM, sphingomyelin; DSM, dihydrosphingomyelin; LysoPE, lysophosphatidylethanolamine; ppm, parts per million. See Table S3 for the number of fish in each treatment. Detailed information regarding *p*-values and fold changes for all lipids can be found in Table S8.

	Control versus Dose Trend				
Lipid Group	188 ppm	242 ppm	253 ppm	294 ppm	375 ppm
PE(34:1)	-	-	-	Decrease	-
PE(36:5)	Decrease	-	-	-	-
PS(34:1)	-	-	-	Decrease	Decrease *
PS(38:4)	-	-	Decrease	-	Decrease
PS(40:5)	-	-	-	Decrease	-
PS(42:6)	Decrease	-	Decrease	-	Decrease
PS(42:9)	Decrease	-	-	Decrease *	Decrease
PS(44:12)	Decrease	-	-	-	-
Total PS	Decrease	-	-	-	-
ePE(36:1)	-	-	-	Decrease	-
Total ePC	-	-	-	Decrease *	Decrease *
Total LysoPE	-	-	-	Decrease	-
DSM(16:0)	-	-	-	-	Decrease *
Total SM and DSM	-	-	-	Decrease	-

* *p*-value < 0.01, No color indicates fold a fold change between 0 and 1, orange color indicates fold changes between 1 and 2, red color indicates fold changes greater than 2.

**Table 4:** Summary of PFHxS significant trends found using Tukey HSD on the lipidomics endpoints. Decrease or Increase indicates trend of accumulation of lipid group in control vs. treated sample. All lipid species shown in the table were significantly different in treated samples vs. the control at a *p*-value < 0.05 and an * indicates lipid species significant a *p*-value < 0.01. - indicates no significant pattern in lipid group accumulation in treated sample vs. control. See Fahy et al. (2009) [65] for detailed lipid acronym definition. PE, phosphatidylethanolamine; PC, phosphatidylcholine; PS, phosphatidylserines; ePC, ether-phosphatidylcholine; ppm, parts per million. See Table S3 for the number of fish in each treatment. Detailed information regarding *p*-values and fold changes for all lipids can be found in Table S8.

	Control verses Dose Trend				
Lipid Group	6.4 ppm	8.7 ppm	10.2 ppm	11.5 ppm	14.4 ppm
ePC(34:1)	-	-	-	- ^1^	Decrease ^1^
ePC(36:2)	-	-	-	- ^1^	Decrease *^,1^
Total ePC	-	-	-	- ^1^	Decrease ^1^
PC(32:1)	-	-	-	- ^1^	Decrease ^1^
PC(34:3)	-	-	-	- ^1^	Decrease ^1^
PE(36:5)	-	-	-	- ^1^	Decrease ^1^
PE(46:12)	-	-	-	- ^1^	Decrease ^1^
PS(38:4)	-	-	Decrease	- ^1^	Decrease ^1^
PS(40:5)	-	-	-	- ^1^	Decrease ^1^

^1^ These results were from a low number of embryos, * *p*-value < 0.01, No color indicates a fold change between 0 and 1, orange color indicates fold changes between 1 and 2, red color indicates fold changes greater than 2.

**Table 5:** Summary of PFOS significant trends found using Tukey HSD on the lipidomics endpoints. Decrease or Increase indicates trend of accumulation of lipid group in control vs. treated sample. All lipid species shown in the table were significantly different in treated samples vs. the control at a *p*-value < 0.05 and an * indicates lipid species significant a *p*-value < 0.01. - indicates no significant pattern in lipid group accumulation in treated sample vs. control. See Fahy et al. (2009) [65] for detailed lipid acronym definition. PE, phosphatidylethanolamine; PS, phosphatidylserines; FA, fatty acid; ePE, ether-phosphatidylethanolamine; PA, phosphatidic acid; ppm, parts per million. See Table S3 for the number of fish in each treatment. Detailed information regarding *p*-values and fold changes for all lipids can be found in Table S8.

	Control verses Dose Trend				
Lipid Group	1.21 ppm	1.40 ppm	1.86 ppm	2.03 ppm	2.42 ppm
FA(16:0)	-	-	-	-	Increase ^1^
FA(18:1)	-	-	-	-	Increase ^1^
FA(18:2)	-	-	-	-	Increase ^1^
FA(20:1)	-	-	-	-	Increase ^1^
FA(20:2)	-	-	-	-	Increase ^1^
FA(20:5)	-	-	-	-	Increase ^1^
FA(22:6)	-	-	-	-	Increase ^1^
PA(34:1)	-	Decrease	Decrease *	Decrease *	Decrease ^1^
PE(32:1)	-	-	-	-	Increase *^,1^
PE(38:3)	-	-	-	-	Decrease ^1^
PE(38:4)	-	-	-	-	Decrease ^1^
PE(38:5)	-	-	-	Decrease	Decrease ^1^
PE(40:8)	-	-	-	-	Decrease ^1^
PE(42:10)	-	-	-	-	Decrease ^1^
PE(42:8)	-	-	-	Decrease	Decrease ^1^
PS(40:5)	-	-	-	Decrease	Decrease ^1^
PS(40:6)	-	-	-	Decrease	Decrease ^1^
Total_PS	-	-	-	-	Decrease ^1^
ePE(40:2)	-	-	-	-	Decrease ^1^
Total_ePE	-	-	-	-	Decrease ^1^

^1^ These results are from embryos of low number and quality, * *p*-value < 0.01, No color indicates a fold change between 0 and 1, orange color indicates fold changes between 1 and 2, red color indicates fold changes greater than 2.

## Data Availability

The original contributions presented in the study are included in the article/supplementary materials, further inquiries can be directed to the corresponding author.

## Supplementary Materials

The following supporting information can be downloaded at: https://www.mdpi.com/article/10.3390/toxics12030192/s1, Figure S1: Plate randomization for each PFAS exposure; Figure S2: PFHxS treatment impacts on zebrafish embryo behaviors that described the reaction response after a visual startle; Figure S3: PFHxS treatment impacts on zebrafish embryo swimming behavior; Figure S4: PFOA treatment impacts on zebrafish embryo behaviors that describe the reaction response after a visual startle; Figure S5: PFOA treatment impacts on zebrafish embryo swimming behavior; Table S1: Number of fish used in the behavior analysis for each chemical treatment; Table S2: Description of behavior endpoints examined in this study; Table S3: Number of embryos in each pooled lipidomics sample; Table S4: Details of lipid standards used in lipidomics analysis, Table S5: Parameters and setting for the XEVO-TQS#WAA627 instrument to determine lipid quantities using in the lipidomics analysis; Table S6: List of lipids examined in this study; Table S7: Results from individual permutation ANOVAs conducted in this study; Table S8: Results from Tukey HSD pairwise comparison tests; Table S9: Significant results from Tukey HSD significant pairwise comparison tests organized to show which endpoint increased (green) or decreased (red) after exposure to various PFAS chemical concentrations; Table S10: Predicted chemical concentrations at 10 and 50% lethality on 120 hpf embryos; Table S11: Model parameters used to estimate lethal dose concentrations.
