# Supplementary material for: Per- and Polyfluoroalkyl Substances: Impacts on Morphology, Behavior and Lipid Levels in Zebrafish Embryos
Source: Toxics. 2024 Feb 29;12(3):192. doi: 10.3390/toxics12030192 (PMC10975676; doi:10.3390/toxics12030192)
Supplement: Supplementary file 1 [file toxics-12-00192-s001.zip › toxics-2801962_Supplementary_Material_2_28_24.pdf]

Supplementary Material

1 Tables

Table S1. Number of fish used in the behavior analysis for each chemical treatment. Each treatment began with 96 embryos.

				Behavior Analysis		
Chemical	Media Concentration (ppm)	Mortality at 96 hpf	Mortality at 120 hpf	Removed due to deformities	Removed due to Tracking Problems	Final Total
PFOA	0	0	0	0	0	88
	188	0	0	0	1	87
	242	1	2	1	1	90
	253	0	2	0	1	93
	294	4	11	0	1	84
	375	6	16	0	1	79
PFHxS	0	2	2	0	0	94
	6.35	1	2	1	0	94
	8.70	0	6	0	0	89
	10.15	1	16	5	0	75
	11.50	0	39	7	0	50
	14.35	2	52	10	1	33
PFOS	0	2	2	NA	NA	NA
	1.21	0	4	NA	NA	NA
	1.40	2	13	NA	NA	NA
	1.86	1	31	NA	NA	NA
	2.03	2	50	NA	NA	NA
	2.42	9	59	NA	NA	NA

Table S2. Description of behavior endpoints examined in this study.

Behavior Endpoint	Definition
Total Distance Traveled (mm)	Total distanced traveled during swimming bouts for the entire 24 minute assay.
Total Time Swimming (sec)	Total time embryo was swimming during 24 minute test.
Overall Step Length (mm)	Per frame distance traveled during a 0.033 second period (one frame to the next) averaged over the entire 24 minute test [i.e. includes zeros when fish moved less than 1 mm/s for more than 5 frames (0.166 sec)].
Overall Step Length Variation	Standard deviation of distance traveled during 0.033 second period (one frame to the next).
Overall Turning Angle	Turning angle during 0.033 second period (one frame to the next) averaged over frames when fish were swimming. Ranges from -3.14 to 3.14, where negative values indicate right turns and positive values indicate left turns.
Overall Turning Angle Variation	Standard deviation of per frame turning angle during 0.033 second period (one frame to the next).
Swimming Bouts (per sec)	The number of active swimming bouts per second. A swimming bout was defined as movement at least 1 mm/s for more than 5 frames (0.166 sec).
Swimming Bout Duration (sec)	Mean duration of all swimming bouts averaged over the 24 minute period.
Swimming Bout Speed (mm/s)	Per frame swimming speed averaged during a swimming bout; average bout speed averaged over the 24 minute period.
Swimming Bout Turning Angle	Per frame absolute turning angle averaged during a swimming bout; average absolute turning angle averaged over the 24 minute period. Ranges from -3.14 to 3.14, where negative values indicate right turns and positive values indicate left turns.
Startle Magnitude (mm/sec)	Maximum velocity within 5 seconds after the startle after subtracting the speed the embryo was swimming at the time of the startle
Startle Response time (sec)	Difference in time between the startle and the maximum distance traveled within 5 seconds after the startle
Startle Distance (mm)	Distance traveled from the time of the startle to the time of startle magnitude
Distance after Startle (mm)	Distance traveled from the time of the startle to 5 seconds after the startle

Table S3. Number of embryos in each pooled lipidomics sample

		Number of Embryos used in Lipid Omics					
Chemical	Plate	0 ppm	188 ppm	242 ppm	253 ppm	294 ppm	375 ppm
PFOA	2	8	8	8	8	6	5
	5	8	8	7	8	7	7
	9	8	8	8	8	7	8
	10	8	8	8	6	6	8
	12	8	8	8	8	7	6
		0 ppm	6.4 ppm	8.7 ppm	10.2 ppm	11.5 ppm	14.4 ppm
PFHxS	4	8	8	6	0	0	0
	5	8	7	7	3	0	0
	6	7	8	8	0	0	0
	11	7	8	4	6	0	0
	1	8	8	8	5	4	3
	2	8	8	8	6	4	2
		0 ppm	1.21 ppm	1.40 ppm	1.86 ppm	2.03 ppm	2.42 ppm
PFOS	1	8	8	7	7	4	3
	2	7	8	7	8	6	2
	3	8	8	8	7	3	3
	4	8	7	7	6	5	3
	5	8	8	7	7	2	2

Table S4. Details of lipid standards used in lipidomics analysis.

Compound Formula	Compound Name	Alternative Name	Mass of Ion	Used to Quantify	Scan Mode	Adduct Detected	nmol/ sample
C21H44O7PN	lysoPC(13:0)		454.3	LysoPC	+Prec184	[M+H]+	0.12
C27H56O7PN	lysoPC(19:0)		538.3	LysoPC	+Prec184	[M+H]+	0.12
C32H64O8PN	PC(12:0/12:0)		622.4	PC	+Prec184	[M+H]+	0.12
C56H108O8PN	PC(24:1/24:1)		954.9	PC	+Prec184	[M+H]+	0.12
C19H40O7PN	lysoPE(14:0)		426.3	LysoPE	+NL141	[M+H]+	0.06
C23H48O7PN	lysoPE(18:0)		482.3	LysoPE	+NL141	[M+H]+	0.06
C29H58O8PN	PE(12:0/12:0)		580.5	PE	+NL141	[M+H]+	0.06
C45H90O8PN	PE(20:0/20:0)	diphytanoyl PE	804.7	PE	+NL141	[M+H]+	0.06
C34H67O10P	PG(14:0/14:0)		684.5	PG	+NL189	[M + NH4]+	0.06
C46H91O10P	PG(20:0/20:0)	diphytanoyl PG	852.7	PG	+NL189	[M + NH4]+	0.06
C34H66O10PN	PS(14:0/14:0)		680.6	PS	+NL185	[M+H]+	0.04
C46H90O10PN	PS(20:0/20:0)	diphytanoyl PS	848.8	PS	+NL185	[M+H]+	0.04
C31H61O8P	PA(14:0/14:0)		610.5	PA	+NL115	[M + NH4]+	0.06
C43H85O8P	PA(20:0/20:0)	diphytanoyl PA	778.7	PA	+NL115	[M + NH4]+	0.06
C43H83O13P	PI(16:0/18:0)		856.70	PI	+NL277	[M + NH4]+	0.06
C15H30O2[a]	15:0 FA		241.20	fatty acids	+MS1	[M + NH4]+	0.20
C15H30O2[b]	15:0 fatty acid		241.2	free fatty acids	MS1	[M-H]-	0.2

[a] for PFAS treatments

[b] for PFOA and PFHxS treatments

Table S5. Parameters and setting for the XEVO-TQS#WAA627 instrument to determine lipid quantities using in the lipidomics analysis. Method a: animalPL-negPG022219.EXP method: continuous infusion from loop, method b: FFA-negMS1-032917.EXP. Constant settings included: Automatic cycle time, source temperature of 150oC, desolvation temperature of 250oC, cone gas flow of 150 L/Hr, desolvation gas flow of 650 L/Hr, nebuliser gas flow of 7 bar, acquisition cone voltage of 40V, and data subtraction of (1, 40, 0.01).

Found in supplementary .xlsx file

Table S6. List of lipids examined in this study.

Found in supplementary .xlsx file

Table S7. Results from individual permutation ANOVAs conducted in this study.

Found in supplementary .xlsx file

Table S8. Results from Tukey HSD pairwise comparison tests.

Found in supplementary .xlsx file

Table S9. Significant results from Tukey HSD significant pairwise comparison tests organized to show which endpoint increased (green) or decreased (red) after exposure to various PFASs chemical concentrations.

Found in supplementary .xlsx file

Table S10. Predicted chemical concentrations at 10 and 50% lethality on 120 hpf embryos.

		Predicted Concentration Lethal to 10% of Embryos (ppm)				Predicted Concentration Lethal to 50% of Embryos (ppm)			
				Confidence Limit				Confidence Limit	
Substrate	Chemical	Mean	Standard Error	Lower	Upper	Mean	Standard Error	Lower	Upper
Media									
	PFOA	318.08	1.47	149.88	675.01	528.64	3.01	60.91	4587.93
	PFHxS	8.55	1.51	3.82	19.15	14.28	1.51	6.34	32.18
	PFOS	1.41	1.43	0.696	2.84	2.14	1.28	1.32	3.46
Tissue									
	PFOA	498.07	3.90	34.55	7180.33	2030.12	883.78	0.00	1208508074.98
	PFHxS	58.83	2.66	8.67	399.16	124.27	3.61	10.05	1536.50
	PFOS[a]	14.05	85.75	0.00	86434.23	849.85	46.35	0.46	1566064.13

[a] This model is uncertain due to high variance between treatments.

Table S11. Model parameters used to estimate lethal dose concentrations.

			Intercept Parameter Estimates				Log_{10}(Media or Tissue Concentration) Parameter Estimates				Model Variance Estimates				
Substrate	Chemical	Best Fit Model	Mean	Std	Z-value	P-value	Mean	Std	Z-value	P-value	Residual Deviance	Residual Deviance df	Null Deviance	Null Deviance df	AIC
Media															
	PFOA	Probit	-15.8178	27.5889	-0.5733	0.5664	2.5227	4.8139	0.5240	0.6003	0.0453	3	0.3758	4	4.6957
	PFHxS	Probit	-6.6528	7.3478	-0.9054	0.3653	2.5022	3.0477	0.8210	0.4116	0.0986	3	0.9262	4	6.8362
	PFOS	Probit	-2.3274	2.0263	-1.1486	0.2507	3.0647	3.0219	1.0142	0.3105	0.0482	3	1.3228	4	7.6247
Tissue															
	PFOA	Probit	-6.9461	22.3648	-0.3106	0.7561	0.9121	3.7384	0.2440	0.8073	0.3160	4	0.4972	5	4.6719
	PFHxS	Probit	-8.2640	21.4975	-0.3844	0.7007	1.7136	4.8928	0.3502	0.7262	0.8047	4	1.3916	5	6.6200
	PFOS[a]	Probit	-2.1070	2.8206	-0.7470	0.4551	0.3124	0.5656	0.5523	0.5807	1.5686	4	1.9945	5	11.4146

[a] This model is uncertain due to high variance between treatments.

2 Figures

Plate 1	B1	B2	B3	B4	B5	B6	B7	B8
Control								
C1								
C2								
C3								
C4								
C5								

Plate 2	B8	B1	B2	B3	B4	B5	B6	B7
C5								
Control								
C1								
C2								
C3								
C4								

Plate 3	B7	B8	B1	B2	B3	B4	B5	B6
C4								
C5								
Control								
C1								
C2								
C3								

Plate 4	B6	B7	B8	B1	B2	B3	B4	B5
C3								
C4								
C5								
Control								
C1								
C2								

Plate 5	B5	B6	B7	B8	B1	B2	B3	B4
C2								
C3								
C4								
C5								
Control								
C1								

Plate 6	B4	B5	B6	B7	B8	B1	B2	B3
C1								
C2								
C3								
C4								
C5								
Control								

Plate 7	B3	B4	B5	B6	B7	B8	B1	B2
Control								
C1								
C2								
C3								
C4								
C5								

Plate 8	B2	B3	B4	B5	B6	B7	B8	B1
C5								
Control								
C1								
C2								
C3								
C4								

Plate 9	B8	B1	B2	B3	B4	B5	B6	B7
C4								
C5								
Control								
C1								
C2								
C3								

Plate 10	B7	B8	B1	B2	B3	B4	B5	B6
C3								
C4								
C5								
Control								
C1								
C2								

Plate 11	B5	B6	B7	B8	B1	B2	B3	B4
C2								
C3								
C4								
C5								
Control								
C1								

Plate 12	B4	B5	B6	B7	B8	B1	B2	B3
C1								
C2								
C3								
C4								
C5								
Control								

Figure S1. Plate randomization for each PFAS exposure. B1 – B8 in the column headers represent the 8 independent breeds used in the 3 PFAS exposures. In the columns, Control = E2 Media control used for each of 3 exposures and C1-C5 represent the PFAS exposure concentrations with increasing number = to increasing PFAS concentration.

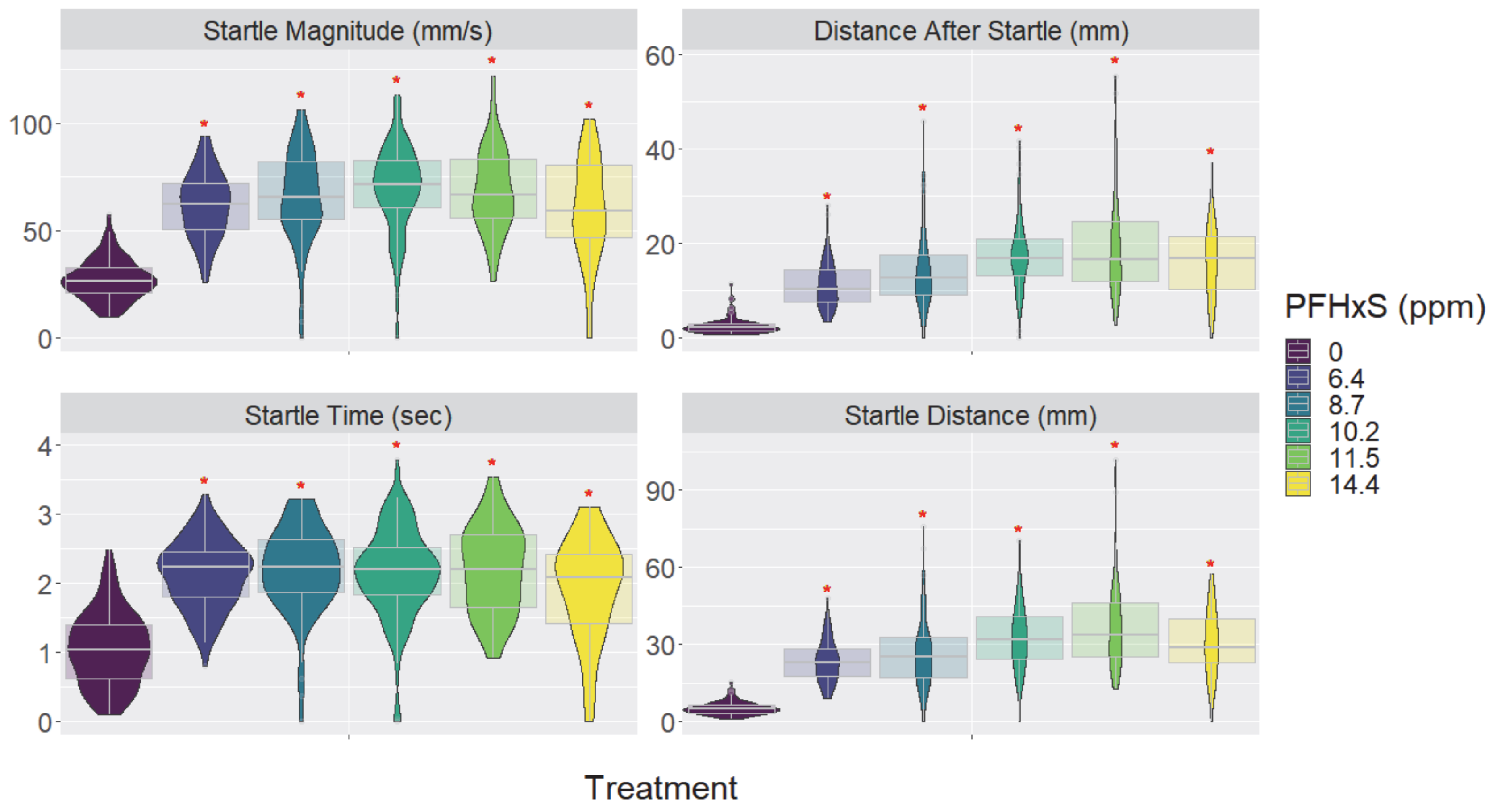

Figure S2. PFHxS treatment impacts on zebrafish embryo behaviors that described the reaction response after a visual startle. PFHxS concentrations are from the media used in each treatment. Red asterisk indicates significant difference between treatment and control groups. Data are presented as violin plots overlaid with the 95th percentile box plot.

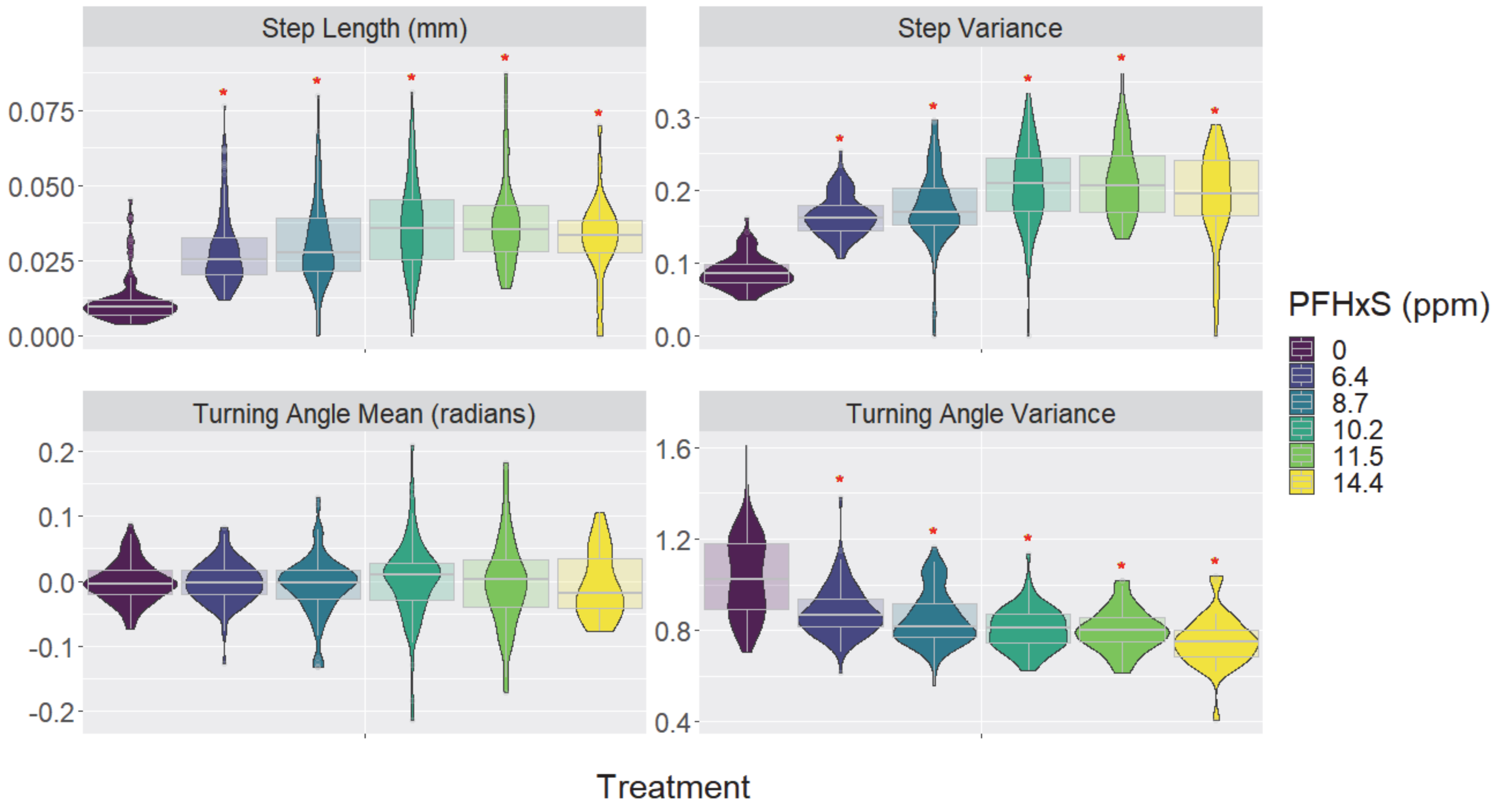

Figure S3. PFHxS treatment impacts on zebrafish embryo swimming behavior. PFHxS concentrations are from the media used in each treatment. Red asterisk indicates significant difference between treatment and control groups. Data are presented as violin plots overlaid with the 95th percentile box plot.

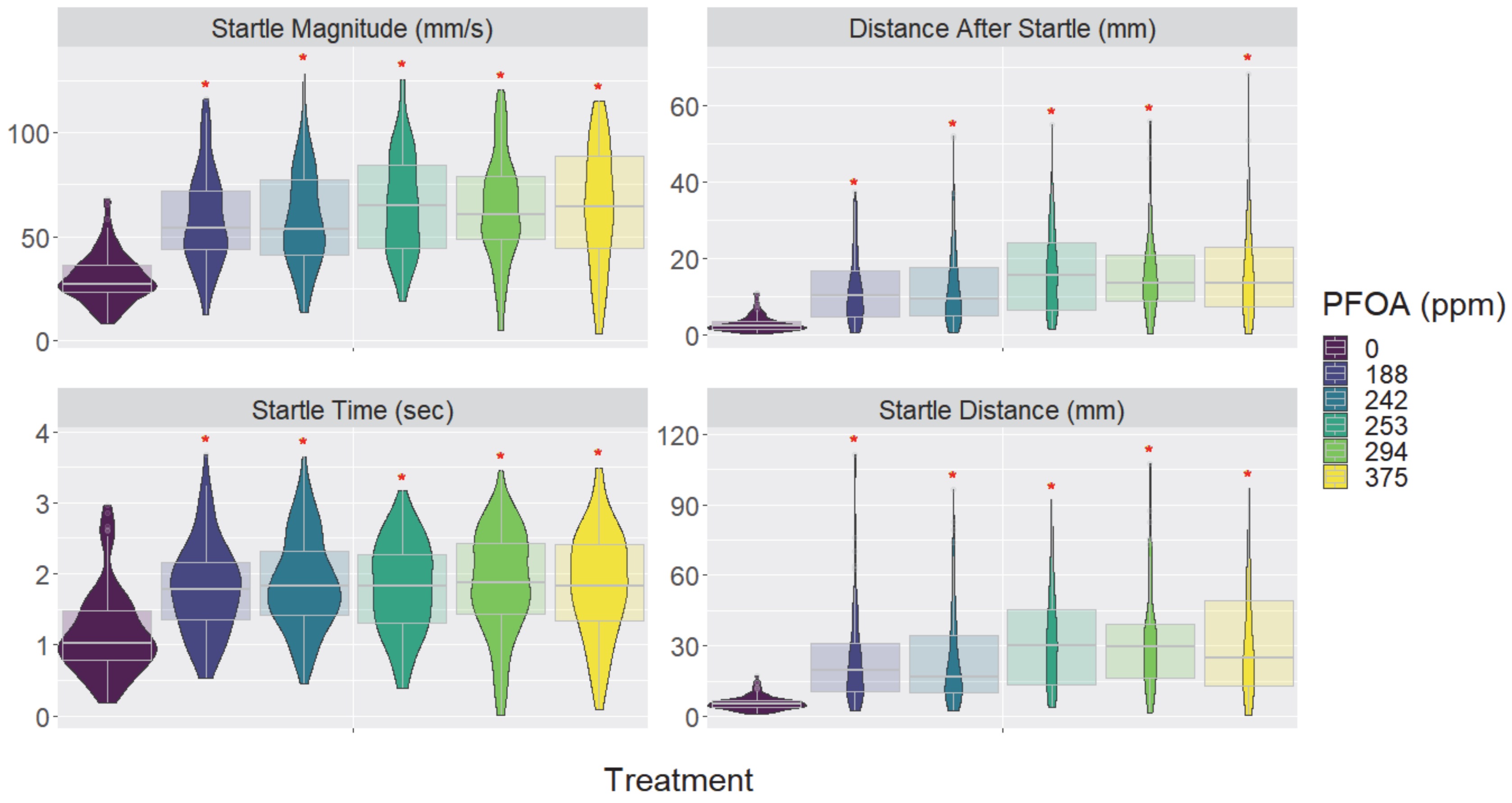

Figure S4. PFOA treatment impacts on zebrafish embryo behaviors that describe the reaction response after a visual startle. PFOA concentrations are from the media used in each treatment. Red asterisk indicates significant difference between treatment and control groups. Data are presented as violin plots overlaid with the 95th percentile box plot.

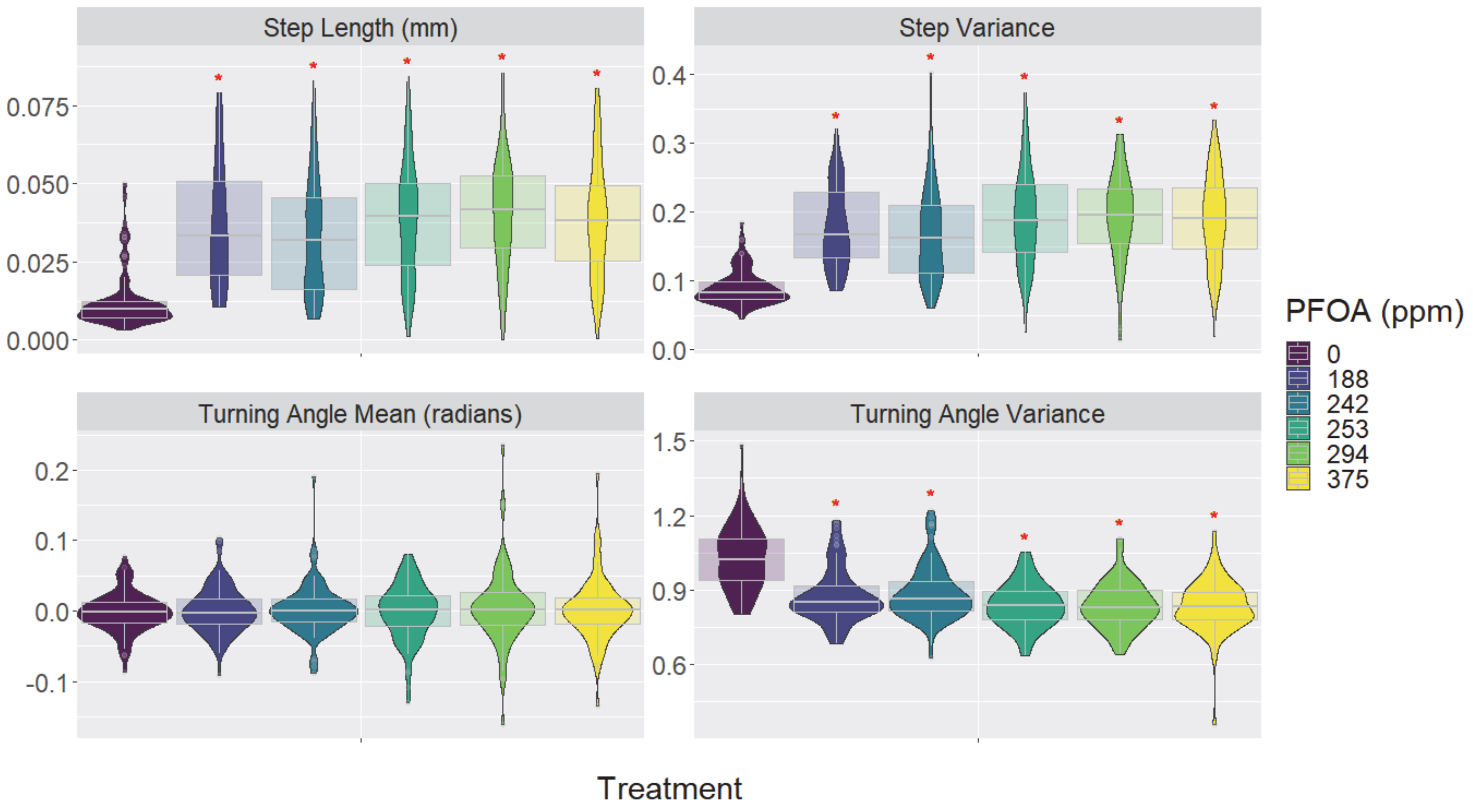

Figure S5. PFOA treatment impacts on zebrafish embryo swimming behavior. PFOA concentrations are from the media used in each treatment. Red asterisk indicates significant difference between treatment and control groups. Data are presented as violin plots overlaid with the 95th percentile box plot.
